# Upregulation of the transcription elongation factor ELL2 by Tax as a novel mechanism of HTLV-1 gene expression

**DOI:** 10.1186/1742-4690-11-S1-O48

**Published:** 2014-01-07

**Authors:** Melanie C Mann, Sarah Strobel, Bernhard Fleckenstein, Andrea K Kress

**Affiliations:** 1 Institute of Clinical and Molecular Virology, Friedrich-Alexander-Universität Erlangen-Nürnberg, Erlangen, Germany

## 

During HTLV-1 gene expression, positive transcription elongation factor b (pTEFb) is recruited to the long terminal repeat by Tax. Usually, additional proteins and elongation factors, known as the super elongation complex (SEC) and acting together with pTEFb, are required for efficient transcriptional elongation. To decipher further Tax targets that play a role in transcriptional elongation, microarray analysis was performed. Among all known cellular elongation factors, the eleven nineteen lysine-rich elongation factor 2 (ELL2) was the only one selectively upregulated in the presence of HTLV-1/Tax. ELL2 is known as the stoichiometrically limiting factor of the SEC. Further analysis of various HTLV-1-transformed and patient-derived cell lines by qPCR and immunoblot revealed ELL2 to be significantly upregulated in the presence of Tax. Repression of Tax in Tax-transformed Tesi cells leads to reduced amounts of ELL2 mRNA and protein. Moreover, siRNA-mediated knockdown of Tax in MT-2 diminishes ELL2 expression. Transfection of increasing amounts of Tax in 293T cells leads to an increase of ELL2 transcripts, suggesting that ELL2 expression is dependent on Tax. Furthermore, we found that coexpression of ELL2 significantly increases Tax-mediated transactivation of the HTLV-1 promotor in a dose-dependent manner. The enhanced transactivation is likely due to a promotor-independent and Tax-specific enhancement of Tax expression by ELL2. Interestingly, siRNA-mediated knockdown of ELL2 in MT-2 leads to strong reduction of Tax protein, suggesting a positive feedback loop between ELL2 and Tax in HTLV-1-infected cells. Taken together, we identified ELL2 as a new factor which may play an important role in HTLV-1 transcription.

